# Supplementary material for: Social Interactions in Two Groups of Zoo-Housed Adult Female Asian Elephants (Elephas maximus) that Differ in Relatedness
Source: Animals (Basel). 2018 Aug 1;8(8):132. doi: 10.3390/ani8080132 (PMC6115912; doi:10.3390/ani8080132)
Supplement: Supplementary file 1 [file animals-08-00132-s001.zip › animals-329114-SI.pdf]

Supplementary material

Social Interactions in Two Groups of Zoo-Housed Adult Female Asian Elephants (*Elephas Maximus*) that Differ in Relatedness

Naomi D. Harvey, Carolyn Daly, Natasha Clark, Eleanor Ransford, Stefanie Wallace and Lisa Yon *

School of Veterinary Medicine and Science, The University of Nottingham, Leicestershire, LE12 5RD, UK; Naomi.Harvey@nottingham.ac.uk (N.D.H.); svycad@nottingham.ac.uk (C.D.); svync2@nottingham.ac.uk (N.C.); svyer1@nottingham.ac.uk (E.R.); Stefanie Wallace (svysaw@nottingham.ac.uk (S.W.)

* Correspondence: Lisa.Yon@nottingham.ac.uk; Tel.: +44-115-951-6358

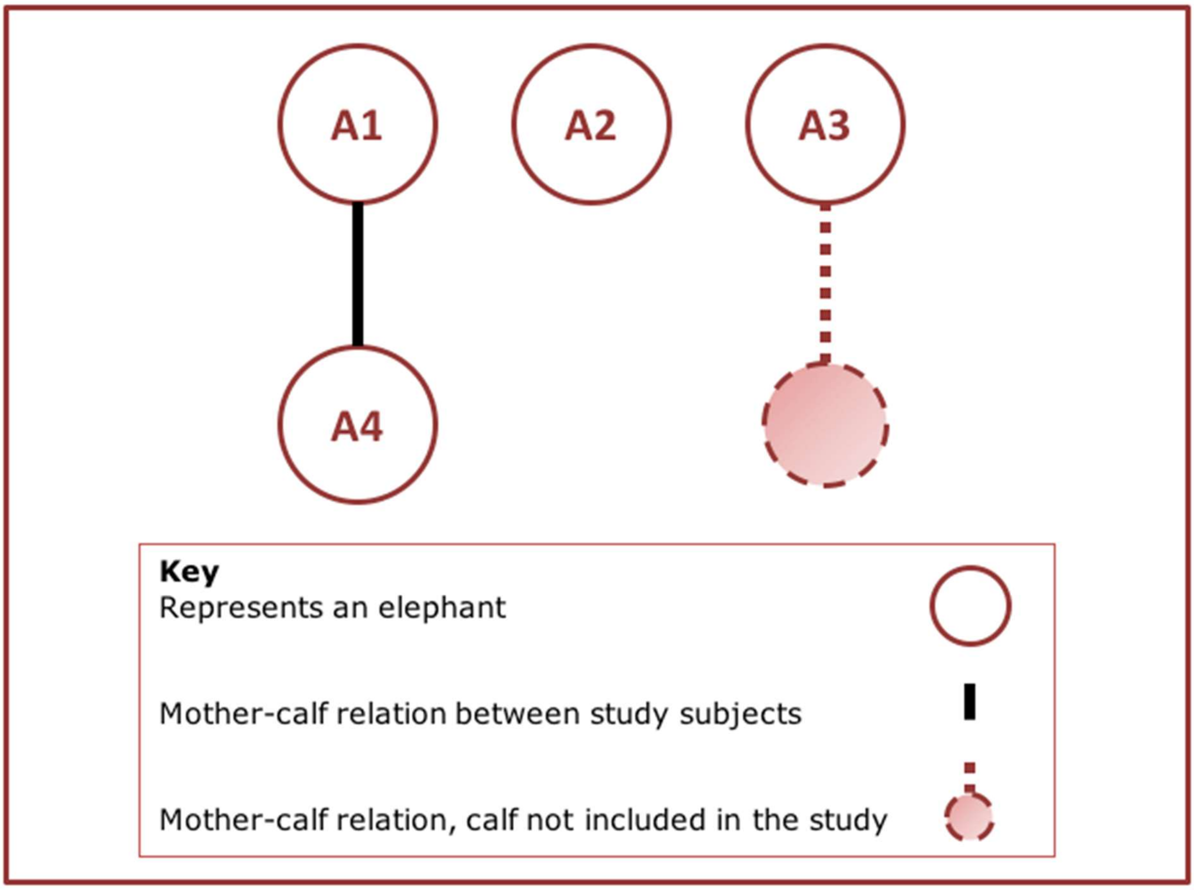

Figure S1. Visual depiction of group relatedness at Zoo A. A1–A4 represent the study subjects. A1 and A4 were mother and daughter. A3's calf, was not included in the study but is indicated on the diagram.

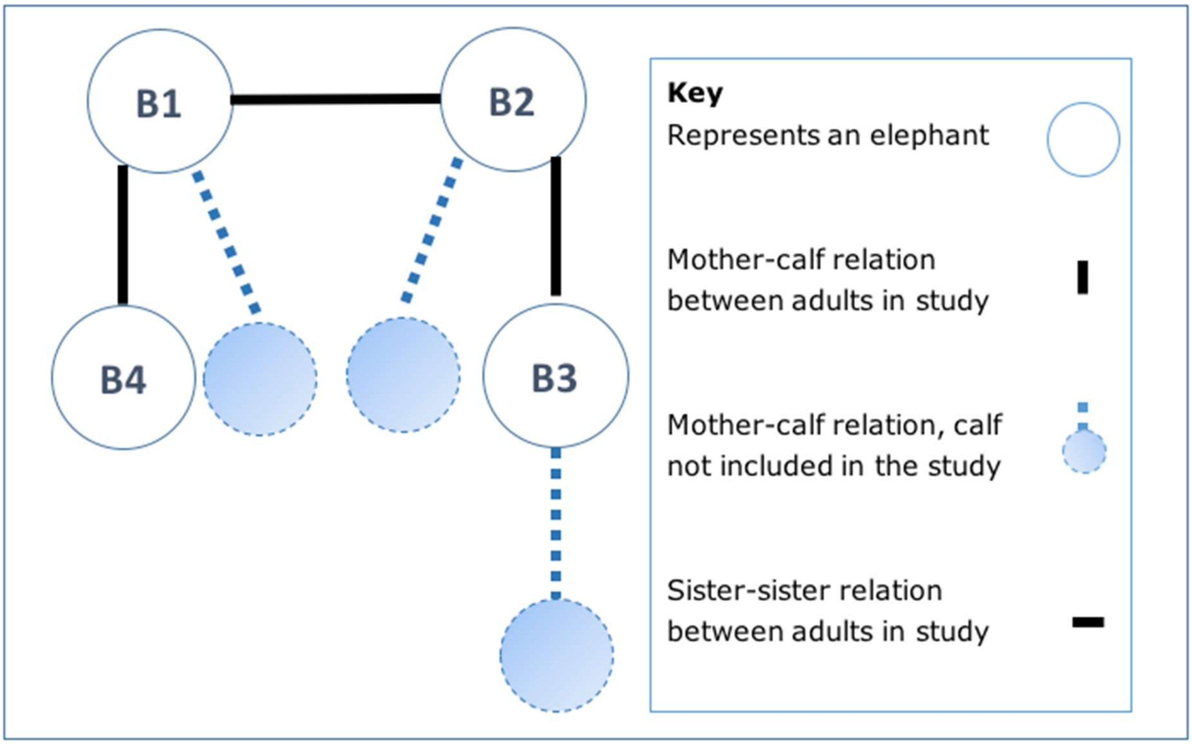

Figure S2. Visual depiction of group relatedness at Zoo B. B1-B4 represent the study subjects. B1 and B2 were full sisters and B4 and B3 their daughters, meaning that B4 and B3 are cousins. The three calves not included in this study are indicated on the diagram.

Table S1. Original social behaviour ethogram tested during live observation pilots at both study Zoo's.

Valence	Behaviour	Code	Descriptions
Affiliative	Leaning	Lean	Leaning on another elephant
Affiliative	Lying interaction	Ly	Standing over another elephant - usually one that is lying down or young; Placing at least one foot on top of another elephant - usually one that is lying down; Sitting in a crouched position on top of another elephant which is in lying rest
Affiliative	Trunk touch	TT	Putting the trunk in the mouth of another elephant; Touching another elephant, not the mouth or genitals, with the trunk in a non-aggressive manner; Touching the genital area of another elephant with the trunk;
Affiliative	Trunk hold	TH	Intertwining of trunks between two elephants
Affiliative	Body rub or Nudge	BR/N	Gentle physical contact between two elephants which may be head-head, head-body or body-body (not including touching with trunk); Rubbing the body against another elephant
Affiliative	Approach	Ap	Moving in a non-aggressive manner, within one body length of another elephant
Affiliative	Parallel walk	PW	Two elephants walking side by side in a non-aggressive manner, for 3 or more steps
Affiliative	Follow	F	One elephant walks closely behind (within 2 elephant body lengths) of another elephant
Affiliative	Offer food	Feed	One elephant pushes a pile of food towards another elephant, looks like an offering of the resource

Affiliative	Trunk Lift	TL	Trunk is outstretched and raised towards an approaching individual
Affiliative	Play	Play	Engaging in active play with another elephant, including head to head sparring, trunk wrestling, mounting, chasing, and rolling on one another. Does not include behaviour observed following an antagonistic encounter or as part of courtship
Agonistic	Kick or hit	K/H	Strike out or hit an elephant with a foot in a seemingly aggressive manner - may include kicking of sand towards another elephant; Hitting another elephant with the trunk or tail
Agonistic	Charge	Charge	Move towards another elephant with the head held high, pace usually quickens as individual gets closer to the target elephant
Agonistic	Chase	Chase	Charge leading to pursuit of another elephant
Agonistic	Push	Push	One elephant forces or pushes against the body (usually the rump) of another elephant, resulting in the elephant that is being pushed moving at least two steps
Agonistic	Stand off	SO	Two elephants standing facing in opposite directions with foreheads pushing against each other
Agonistic	Floor smack	FS	Hitting the trunk on the floor in an aggressive manner, may be accompanied by a 'snort'
Agonistic	Lunge	Lunge	A lunging motion followed by physical contact, used to prevent another elephant standing up
Agonistic	Tusking	Tusk	Poking or jabbing at another elephant with the tusk
Agonistic	Directed trunk swing	TS	head oriented towards another elephant, human or change in the environment, violently swinging the trunk around in an aggressive display
Agonistic	Displacement	Dis	Movement of one elephant resulting in another elephant leaving its location (within 10 seconds)
Agonistic	Aggressive display: standing	AggS	Facing another elephant in an aggressive posture; head held high, ears wide or flapping
Agonistic	Aggressive display: walking	AggW	Facing another elephant while walking with head bobbing up and down or side to side, ears wide or flapping
Agonistic	Size up	SU	Two elephants directly facing each other, standing as tall as possible, heads raised, and ears spread wide
Agonistic	Bite	Bite	Biting of the body, trunk or tail of another elephant

Table S2. Asymmetric matrices for each zoo used to create weighted digraphs for each social behaviour classification: affiliative (a1,2); agonistic (b1,2); and non-contact displacement (c1,2). Values were formed by combining the 5 hour adjusted interaction sums from the Spring and Autumn observations to represent 10 hours of observations.

(a1) Zoo A: 10h Affiliative Matrix

Initiator	Receiver			
	A2	A3	A1	A4
A2	-	17	26	8
A3	8	-	32	12
A1	10	3	-	12
A4	5	2	10	-

(b1) Zoo A: 10h Agonistic Matrix

Initiator	Receiver			
	A2	A3	A1	A4
A2	-	5	13	6
A3	1	-	36	6
A1	0	1	-	1
A4	0	0	0	-

(c1) Zoo A: 10h Non-contact Displacement Matrix

Initiator	Receiver			
	A2	A3	A1	A4
A2	-	8	2	3
A3	0	-	1	6
A1	0	1	-	0
A4	0	0	0	-

(a2) Zoo B: 10h Affiliative Matrix

Initiator	Receiver			
	B1	B2	B4	B3
B1	-	5	14	8
B2	13	-	12	9
B4	34	31	-	24
B3	6	23	26	-

(b2) Zoo B: 10h Agonistic Matrix

Initiator	Receiver			
	B1	B2	B4	B3
B1	-	1	2	3
B2	8	-	3	1
B4	2	0	-	1
B3	0	3	2	-

(c2) Zoo B: 10h Non-contact Displacement Matrix

Initiator	Receiver			
	B1	B2	B4	B3
B1	-	2	5	16
B2	19	-	10	2
B4	3	2	-	1
B3	1	1	3	-

Table S3. Minutes in view, strength and strength per 5 hours for each social behaviour classification, for each elephant, at each time point. Aff = affiliative. Ago = agonistic. NCD = non-contact displacement.

ID	Beh	Spring			Autumn		
		**Min. in View**	**Strength**	**5hr Strength**	**Min. in View**	**Strength**	**5hr Strength**
A1	Aff	502	21	13	438	17	12
A2	Aff	547	26	14	350	43	37
A3	Aff	397	53	40	364	15	12
A4	Aff	539	12	7	312	11	11
B1	Aff	600	34	17	571	18	9
B2	Aff	558	32	17	489	27	17
B3	Aff	516	29	17	514	66	39
B4	Aff	544	62	34	467	85	55
A1	Ago	502	3	2	438	0	0
A2	Ago	547	27	15	350	11	9
A3	Ago	397	19	14	364	34	28
A4	Ago	539	0	0	312	0	0
B1	Ago	600	7	4	571	3	2
B2	Ago	558	16	9	489	6	4
B3	Ago	516	4	2	514	5	3
B4	Ago	544	3	2	467	2	1
A1	NCD	502	1	1	438	0	0
A2	NCD	547	7	4	350	10	9
A3	NCD	397	4	3	364	5	4
A4	NCD	539	0	0	312	0	0
B1	NCD	600	17	9	571	28	15
B2	NCD	558	20	11	489	34	21
B3	NCD	516	7	4	514	2	1
B4	NCD	544	10	6	467	1	1

Table S4. The number and type of social agonistic and affiliative social interactions directed towards elephant A1 by A3 in Zoo A.

Period	Affiliative	Agonistic
Spring	2 follows; 16 trunk touches; 8 tail holds; 4 leans; 2 body rubs or nudges [Total = 32]	1 kick/hit; 3 pushes; 1 bite; 12 Other: repetitive nudges [Total = 17]
Autumn	1 follow; 9 trunk touches [Total = 10]	3 kick/hits; 2 pushes; 1 lunge; 2 tail pulls; 1 block; 19 Other: repetitive nudges [Total = 28]

Table S5. Mean hourly rate for affiliative interactions between all possible dyads of captive female Asian elephants from this study plus two others where data was available: Makecha et al. (2012) [1] where data was given in rates per 1 hour, and Coleing (2009) [2] where data was given as frequencies from 40 hours of observation, which was calculated down to represent a 1-hour rate. *indicates a dyad containing a juvenile.

Study	Dyad	Elephant 1	Elephant 2	Hourly rate	Related?
Makecha	CA:KA	0.27	0.9	1.17	Yes
Coleing	THW:SITHAMI	0	82	2.05	Yes
Coleing	THW:SUNDARA *	0	0	0	Yes
Coleing	SUN:SITH *	110	84	4.85	Yes
Harvey	A1:A4	10	12	2.2	Yes
Harvey	B1:B2	5	13	1.8	Yes
Harvey	B1:B3 *	8	6	1.4	Yes
Harvey	B1:B4 *	14	34	4.8	Yes
Harvey	B2:B3 *	9	23	3.2	Yes
Harvey	B2:B4 *	12	31	4.3	Yes
Harvey	B3:B4 *	26	24	5	Yes
Makecha	ME:RO	2.36	1.37	3.73	No
Makecha	ME:KA	0.84	2.58	3.42	No
Makecha	ME:SI	1.18	0.34	1.52	No
Makecha	ME:TI	0.21	0.27	0.48	No
Makecha	ME:CA	0.1	0.03	0.13	No
Makecha	TI:KA	1.9	1.72	3.62	No
Makecha	SI:KA	0.37	0.34	0.71	No
Makecha	KA:RO	0.9	0.64	1.54	No
Makecha	RO:CA	0.2	0.05	0.25	No
Makecha	RO:TI	0.17	1	1.17	No
Makecha	RO:SI	0.05	0.35	0.4	No
Makecha	CA:TI	0.01	0.08	0.09	No
Makecha	CA:SI	0.01	0.14	0.15	No
Coleing	THW:JAN	0	0	0	No
Coleing	THW:SHEBA	0	0	0	No
Coleing	THW:MAYA	0	0	0	No
Coleing	THW:BIRMA	0	0	0	No
Coleing	SUN:JAN *	66	0	1.65	No
Coleing	SUN:MAYA *	0	0	0	No
Coleing	SUN:BIRMA *	0	0	0	No
Coleing	SUN:SHEBA *	0	0	0	No
Coleing	JANG:MAYA	0	0	0	No
Coleing	JANG:BIRMA	0	0	0	No
Coleing	JANG:SHEBA	0	0	0	No
Coleing	JANG:SITH	0	0	0	No
Coleing	MAYA:SITH	0	0	0	No
Coleing	MAYA:SHEBA	0	0	0	No
Coleing	MAYA:BIRMA	0	0	0	No
Coleing	BIRMA:SHEBA	88	76	4.1	No
Coleing	BIRMA:SITH	0	0	0	No
Coleing	SITH:SHEBA	0	0	0	No
Harvey	A1:A2	10	26	3.6	No
Harvey	A1:A3	3	32	3.5	No
Harvey	A2:A3	17	8	2.5	No
Harvey	A2:A4	8	5	1.3	No
Harvey	A3:A4	12	2	1.4	No

Table S6. Mean hourly rate of agonistic interactions between all possible dyads of captive female Asian elephants from this study plus two others where data was available: Makecha et al. (2012) [1] where data was given in rates per 1 hour, and Coleing (2009) [2] where data was given as frequencies from 40 hours of observations, which was calculated down to represent a 1-hour rate. *indicates a dyad containing a juvenile.

Study	Dyad	Elephant 1	Elephant 2	Hourly rate	Related?
Makecha	CA:KA	0	0	0	Yes
Coleing	THW:SITHAMI	0	0	0	Yes
Coleing	THW:SUNDARA *	0	0	0	Yes
Coleing	SUN:SITH *	0	0	0	Yes
Harvey	A1:A4	1	0	0.1	Yes
Harvey	B1:B2	1	8	0.9	Yes
Harvey	B1:B3 *	3	0	0.3	Yes
Harvey	B1:B4 *	2	2	0.4	Yes
Harvey	B2:B3 *	3	1	0.4	Yes
Harvey	B2:B4 *	3	0	0.3	Yes
Harvey	B3:B4 *	2	1	0.3	Yes
Makecha	ME:RO	1.76	0.15	1.91	No
Makecha	ME:KA	0.89	0	0.89	No
Makecha	ME:SI	0.35	0.01	0.36	No
Makecha	ME:TI	0	0	0	No
Makecha	ME:CA	0.07	0	0.07	No
Makecha	TI:KA	0.04	0.01	0.05	No
Makecha	SI:KA	0	0.01	0.01	No
Makecha	KA:RO	0.21	0.2	0.41	No
Makecha	RO:CA	0.12	0	0.12	No
Makecha	RO:TI	0	0.2	0.2	No
Makecha	RO:SI	0.01	0.28	0.29	No
Makecha	CA:TI	0	0	0	No
Makecha	CA:SI	0	0	0	No
Coleing	THW:JAN	3	0	0.075	No
Coleing	THW:SHEBA	0	4	0.1	No
Coleing	THW:MAYA	0	1	0.025	No
Coleing	THW:BIRMA	0	0	0	No
Coleing	SUN:JAN *	0	2	0.05	No
Coleing	SUN:MAYA *	0	0	0	No
Coleing	SUN:BIRMA *	0	0	0	No
Coleing	SUN:SHEBA *	0	0	0	No
Coleing	JANG:MAYA	0	16	0.4	No
Coleing	JANG:BIRMA	2	0	0.05	No
Coleing	JANG:SHEBA	18	0	0.45	No
Coleing	JANG:SITH	2	0	0.05	No
Coleing	MAYA:SITH	1	0	0.025	No
Coleing	MAYA:SHEBA	0	20	0.5	No
Coleing	MAYA:BIRMA	1	0	0.025	No
Coleing	BIRMA:SHEBA	0	0	0	No
Coleing	BIRMA:SITH	0	0	0	No
Coleing	SITH:SHEBA	0	0	0	No
Harvey	A1:A2	0	13	1.3	No
Harvey	A1:A3	1	36	3.7	No
Harvey	A2:A3	5	1	0.6	No
Harvey	A2:A4	6	0	0.6	No
Harvey	A3:A4	6	0	0.6	No

References

1. Makecha, R.; Fad, O.; Gardens, B.; Stan, U.S.A.; Ii, A.K. The Role of Touch in the Social Interactions of Asian Elephants (*Elephas maximus*). *Int. J. Comp. Psychol.* **2012**, *25*, 60–82.
2. Coleing, A. The application of social network theory to animal behaviour. *Biosci. Horiz.* **2009**, *2*, 32–43, doi:10.1093/biohorizons/hzp008.

© 2018 by the authors. Licensee MDPI, Basel, Switzerland. This article is an open access article distributed under the terms and conditions of the Creative Commons Attribution (CC BY) license (http://creativecommons.org/licenses/by/4.0/).
